# A randomized, double-blind, single-dose study (LAVENDER) to assess the safety, tolerability, pharmacokinetics, and immunogenicity of a combined infusion of ABP 980 and pertuzumab in healthy subjects

**DOI:** 10.1007/s00280-021-04334-x

**Published:** 2021-08-05

**Authors:** Vladimir Hanes, Vincent Chow, Tina Stewart, Adeep Puri

**Affiliations:** 1 Amgen Inc., Thousand Oaks, CA USA; 2 Hammersmith Medicines Research Limited (HMR), Cumberland Avenue, London, NW10 7EW UK

**Keywords:** ABP 980, Trastuzumab, Pertuzumab, Co-infusion, Safety, Pharmacokinetics, Biosimilars

## Abstract

**Purpose:**

ABP 980 (KANJINTI^™^) is a biosimilar to reference product HERCEPTIN^®^ (trastuzumab RP). The goal of this study was to characterize the safety, tolerability, and immunogenicity of ABP 980 plus pertuzumab (PERJETA^®^) when co-administered in a single infusion bag in healthy subjects.

**Methods:**

This randomized, double-blind, single-dose, 2-arm, parallel-group study (LAVENDER Study) evaluated an intravenous (IV) infusion of ABP 980 (6 mg/kg) plus pertuzumab (420 mg) combined in a single infusion bag relative to an IV infusion of trastuzumab RP (6 mg/kg) plus pertuzumab (420 mg) combined in a single infusion bag given over 60 min. The subjects were followed for 92 days post dosing.

**Results:**

A total of 42 subjects were enrolled in the study and treated with investigational product. Due to an operational issue during dosing, the first 6 subjects enrolled in the study were replaced. A total of 36 randomized subjects, *n* = 18 for ABP 980 plus pertuzumab and *n* = 18 for trastuzumab RP plus pertuzumab, were treated. Resulting serum concentrations of ABP 980 and trastuzumab RP were similar. There were no serious adverse events, no deaths, and no cardiac disorders during the study. No subject developed anti-drug antibodies throughout the study.

**Conclusions:**

This study demonstrated the safety and tolerability of ABP 980 and pertuzumab admixture in a single infusion bag. The safety profiles and pharmacokinetic parameters of ABP 980 and pertuzumab were consistent with what is known for trastuzumab RP and pertuzumab.

**Clinical trial listing:**

EudraCT 2018-002903-33.

## Introduction

ABP 980 (KANJINTI^®^, Amgen Inc., Thousand Oaks, CA) is a biosimilar to Herceptin^®^ (trastuzumab reference product [RP]; licensed as Herceptin^®^ in the United States [US] and European Union [EU]), a monoclonal antibody (mAb) targeting human epidermal growth factor receptor 2 (HER2) [1–3]. HER2 is overexpressed in approximately 20–30% of breast cancers and gastric cancers, and overexpression is correlated with a worsened prognosis when compared to patients without HER2 amplification. Trastuzumab RP plus chemotherapy is the standard of care for patients with HER2-positive breast cancers and approved in many countries for the treatment of metastatic breast cancer, early breast cancer, and metastatic gastric cancer [1, 2, 4–6]*.* Pertuzumab (PERJETA^®^, Genentech, Inc., South San Francisco, CA) is also an antibody that targets HER2 but because it targets a different subdomain of HER2 than trastuzumab, combined dosing results in a synergistic effect on the inhibition and survival of breast cancer cells [7, 8]. In patients with HER2-positive operable breast cancer, rates of invasive-disease-free survival were significantly improved in the trastuzumab RP plus pertuzumab treatment group compared with trastuzumab RP plus placebo [9]. In patients with HER2-positive metastatic breast cancer, pertuzumab added to trastuzumab RP and docetaxel has been shown to significantly prolong both progression-free survival (PFS) and overall survival (OS) with no increase in cardiac events [10, 11].

As combination therapy of trastuzumab RP plus pertuzumab has become the standard of care for first-line treatment of late stage (stage II to stage III) HER2-positive metastatic breast cancer, an admixture of trastuzumab RP plus pertuzumab in a single 250 mL infusion bag is more efficient for patients and caregivers than two separate 250 mL infusions. Prior to clinical evaluation of trastuzumab RP plus pertuzumab in a single infusion bag, the admixture was demonstrated to be physically and chemically stable, the potency of the mixture and the individual mAbs before and after storage were comparable, and no visual differences were observed in the intravenous (IV) bags that contained admixture compared with the IV bags that contained the individual mAb components over the course of the study [12]. The aim of a single infusion is to increase efficiency via combination dosing as an admixture in a single infusion bag instead of consecutive infusions of the two treatments. To support the administration of the admixture in a single infusion bag in human subjects, an analytical compatibility study was performed to compare ABP 980 plus pertuzumab in a single IV bag versus trastuzumab RP plus pertuzumab mixture in a single IV bag containing 0.9% saline solution, to ensure that the mixed combination is physically and chemically stable for IV administration. The physical and chemical stability results were consistent with the previous admixture evaluation of pertuzumab with trastuzumab RP and the mixtures were determined to be physically and chemically stable for up to 24 h at 5 °C or 30 °C.

In this randomized trial (LAVENDER), we assessed the safety and tolerability of ABP 980 and pertuzumab admixture in a single infusion bag. The frequency, type, and severity of adverse events (AEs), the incidence of anti-drug antibodies (ADAs), and pharmacokinetic (PK) parameters were assessed and compared to the known safety profiles for trastuzumab RP and pertuzumab.

## Materials and methods

### Study Design

**Fig. 1:**
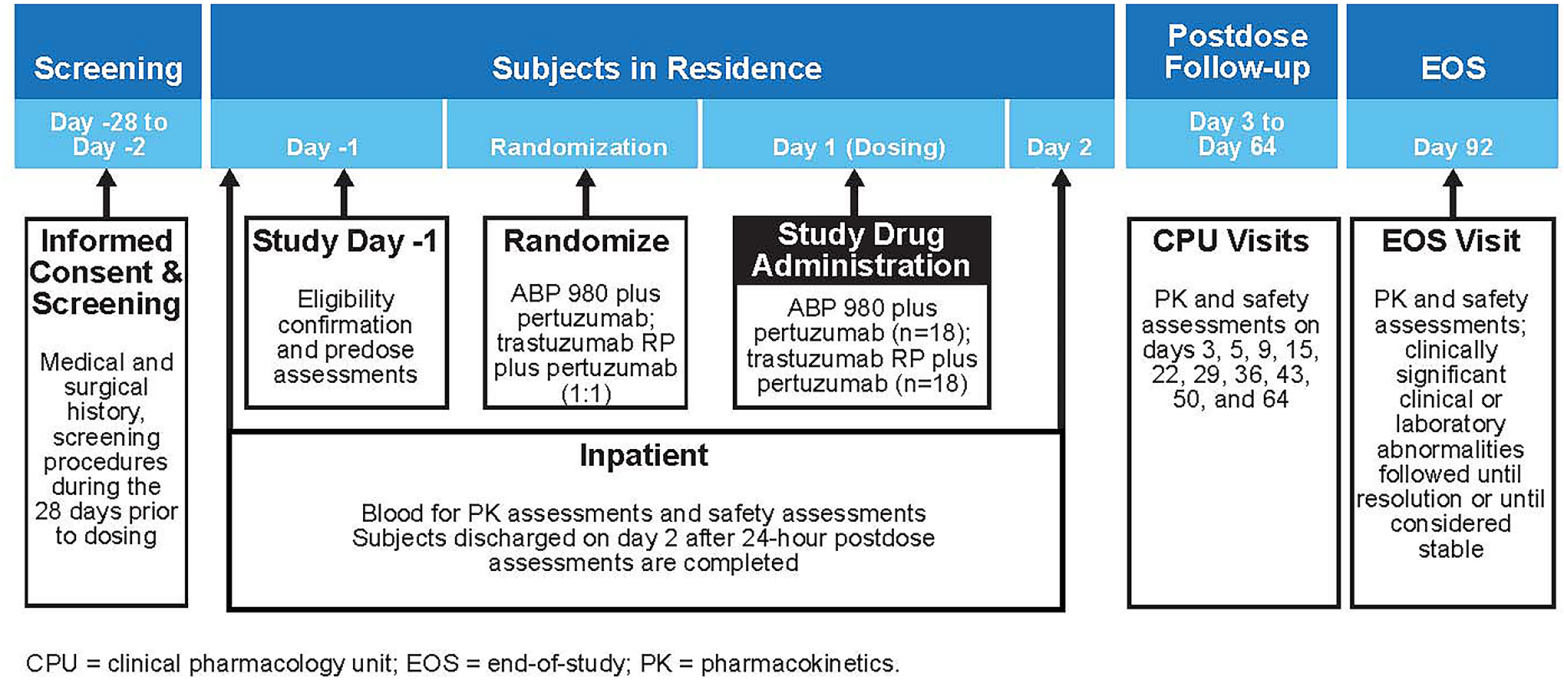
Study design

This trial was a randomized, double-blind, single-dose, 2-arm, parallel-group study in healthy adult male volunteers conducted at a single clinical pharmacology unit (CPU) (Fig. 1). Analyses included a total of 36 healthy male subjects (n = 18 per treatment group) who were randomized 1:1 to receive either an IV infusion of ABP 980 (6 mg/kg) plus pertuzumab (420 mg) combined in a single 250-mL infusion bag or trastuzumab RP (6 mg/kg) plus pertuzumab (420 mg) combined in a single 250-mL infusion bag given over 60 min. Investigational product (IP) was administered on day 1 after predose baseline procedures were completed. Subjects remained in the CPU for at least 24 h after dosing for safety evaluations and PK assessments. They were discharged on day 2 after the 24-h study procedures were completed (at their discretion, investigators could keep subjects in the CPU longer if needed for additional safety monitoring reasons [i.e., potential infusion reaction]). Subjects returned to the CPU on days 3, 5, 9, 15, 22, 29, 36, 43, 50, 64, and 92 (end-of-study [EOS] visit) for safety evaluations and PK assessments. Safety analyses included analyses of AEs and serious adverse events (SAEs), 12-lead ECGs, echocardiograms, local tolerability, and ADAs.

All procedures were performed in accordance with the ethical standards of the institutional and/or national research committee and with the 1964 Helsinki declaration and its later amendments or comparable ethical standards. The final study protocol was approved by an Institutional Review Board and Independent Ethics Committee. 

#### Screening

Study subjects were healthy male adults between 18 and 45 years of age. All subjects were required to meet the study inclusion criteria and provide signed informed consent during the 28 to 2 days prior to dosing. Inclusion criteria included but were not limited to body mass index of 18.0–30.0 kg/m^2^; normal or clinically acceptable physical examination including but not limited to urinalysis, vital signs, and electrocardiogram (ECG); and the ability to communicate effectively with study personnel. Exclusion criteria included but were not limited to men with pregnant partners or not taking measured precautions to prevent pregnancy in partners; history of a clinically significant disorder or condition that could pose a risk to the subject safety or interference with the study; history or presence of conditions known to interfere with the distribution, metabolism, or excretion of drugs; potentially interfering medications or investigational drugs including prior exposure to trastuzumab RP, biosimilars of trastuzumab RP or pertuzumab, or compounds with similar mechanisms of action.

#### Randomization

Subjects were randomized after eligibility was confirmed and predose assessments were completed (Fig. 1). Each subject was tracked by a unique 11-digit subject identification number assigned in sequential order.

#### Investigational products (IPs)

IV infusion bags contained 150 mg of ABP 980 or trastuzumab RP (provided in vials of 21 mg/mL upon reconstitution) and 420 mg of pertuzumab (provided in 14 mL vials of 30 mg/mL each). The IPs were stored in their original container at 2 – 8 °C. Once prepared for IV infusion, ABP 980 plus pertuzumab and trastuzumab RP plus pertuzumab were similar in appearance. The unblinded study site pharmacist matched the appropriate unique infusion bag number to the subject’s randomization group code. Except for an unblinded study monitor if needed, all further study personnel, biostatisticians, and data managers were blinded to treatment assignment for the duration of the study.

### Primary and secondary endpoints

The primary endpoints were safety and immunogenicity. Safety was determined by treatment-emergent AEs as well as clinical laboratory tests, 12-lead ECGs, echocardiograms, physical examinations, local tolerability, and vital signs. The Common Technology Criteria for Adverse Events (CTCAE) classification was used to categorize the severity of AEs. Immunogenicity was determined by the incidence of ADAs. Secondary endpoints were PK parameters (for ABP 980, trastuzumab RP, and pertuzumab), including: area under the serum concentration–time curve from time 0 to infinity (AUC_inf_), maximum observed serum concentration (C_max_), clearance (CL), terminal phase half-life (t_1/2_), time at which the maximum serum concentration was observed (t_max_), terminal elimination rate constant (λ_z_), area under the serum concentration–time curve from time 0 to the time of the last quantifiable concentration (AUC_last_), and last measurable serum concentration (C_last_).

#### Immunogenicity measurements

A 4-mL sample of blood was collected for the measurement of incidence of ADAs on day 1 at baseline prior to dosing and at day 92/EOS. A validated electrochemiluminescence-based bridging immunoassay was used to detect antibodies capable of binding to ABP 980 and which utilized the Meso Scale Discovery platform that followed a two-tiered approach consisting of a screening assay and a specificity assay. Samples were assessed for anti-ABP 980 and anti-trastuzumab RP binding antibodies. Only those samples that tested positive for binding antibodies were further tested for neutralizing antibodies.

#### PK measurements

A 4-mL sample of blood was collected for PK measurement at predose, at 0.5 min before dose, at end of infusion, at 2, 3, 4, 5, 6, 8, and 24 h after the start of the infusion, at each return visit to the CPU (days 3, 5, 9, 15, 22, 29, 36, 43, 50, and 64), and at EOS day 92 (Fig. 1). Serum concentrations were determined using a validated electrochemiluminescent assay.

### Statistical analyses

Descriptive data summaries were tabulated by treatment for all endpoints. Categorical outcomes were summarized by number and percent of subjects falling into each category. The sample size of 18 subjects per arm was considered sufficient to provide descriptive safety data. Assuming a 15% incidence rate for an AE in each arm, the sample size of 18 subjects provides approximately 95% chance of observing at least one AE in each arm. PK parameters were estimated using non-compartmental methods and actual sampling times.

## Results

### Enrollment and disposition of patients

A total of 42 subjects were enrolled in the study and treated with IP. Of the 42 subjects, the first 6 subjects (3 in each treatment group) were replaced and not included in the analysis due to air bubbles forming in the IV line during treatment, which could potentially have influenced the study evaluations. A total of 36 randomized subjects (18 subjects in the ABP 980 plus pertuzumab treatment group and 18 subjects in the trastuzumab RP plus pertuzumab treatment group) were included in all analyses presented here. Demographic and baseline characteristics of subjects are summarized in Table 1 and were similar for the 2 treatment groups. The use of concomitant medications was similar between treatment groups and the most common concomitant medications were paracetamol (9 [50.0%] and 9 [50.0%] subjects in the ABP 980 plus pertuzumab and the trastuzumab RP plus pertuzumab treatment groups, respectively) and ibuprofen (2 [11.1%] and 1 [5.6%] subjects, respectively).

**Table 1:** Demographic and baseline characteristics

	ABP 980 + Pertuzumab (*n* = 18)	Trastuzumab RP + Pertuzumab (*n* = 18)
Race, *n* (%)		
White	10 (55.6)	11 (61.1)
Black or African American	2 (11.1)	3 (16.7)
Asian	2 (11.1)	1 (5.6)
Other	4 (22.2)	2 (11.1)
Age (years), mean (SD)	31.0 (7.5)	28.8 (6.3)
BMI (kg/m^2^), mean (SD)	24.0 (3.0)	23.4 (2.6)

*BMI* body mass index

### Extent of drug exposure

Each of the 36 subjects received the full dose of IP. The mean (± standard deviation [SD]) of the total dose administered of ABP 980 and trastuzumab RP was 444 (52.8) mg and 455 (51.5) mg, respectively. The mean (± SD) total dose of pertuzumab administered was 420 (0.00) mg for both treatment groups. The mean (± SD) duration of infusion was 63.8 (1.11) minutes for the ABP 980 plus pertuzumab group and 63.7 (2.52) minutes for the trastuzumab RP plus pertuzumab group.

### Adverse events

An overall summary of AEs is listed in Table 2. There were no notable differences between treatment groups in the severity of AEs and there were no events greater than grade 2 in severity. No AEs led to withdrawal from the IP or the study. No SAEs or deaths were reported.

**Table 2:** Overall summary of safety results

	ABP 980 + Pertuzumab (*n* = 18) *n* (%) m	Trastuzumab RP + Pertuzumab (*n* = 18) *n* (%) m
Overall adverse events	17 (94.4) 76	17 (94.4) 75
Any grade ≥ 3 or serious adverse events	0	0
Any adverse events leading to discontinuation of study	0	0
Any hypersensitivity adverse events	2 (11.1) 2	1 (11.1) 2
Any infusion reaction adverse events with onset on same date of infusion	5 (27.8) 5	3 (16.7) 3
Headache	10 (55.6) 14	14 (77.8) 17
Nasopharyngitis	3 (16.7) 3	7 (38.9) 9
Diarrhea	5 (27.8) 5	3 (16.7) 3
Feeling cold	4 (22.2) 4	3 (16.7) 3
Pyrexia	4 (22.2) 4	3 (16.7) 4
Dizziness	2 (11.1) 2	3 (16.7) 4
Nausea	2 (11.1) 2	3 (16.7) 4
Vomiting	2 (11.1) 2	3 (16.7) 3

For each category, subjects were included only once, even if they experienced multiple events in that category. Treatment emergence was defined as an adverse event that began or increased in severity or frequency on or after the date of IP administration and up to the EOS visit

*EOS* end-of-study, *IP* investigational product, *m* number of events, *n* number of subjects, *RP* reference product

### Electrocardiogram results

No notable differences between treatment groups were observed for ECG results (Table 3). For subjects who had an abnormal ECG result at any time point, the result was considered not clinically significant. No cardiac disorder AEs were reported for any subject. All subjects had a normal echocardiogram result at baseline and day 92 (EOS). Mean changes in left ventricular ejection fraction were similar for the treatment groups.

**Table 3:** Summary of electrocardiogram results

ECG evaluation time point	ABP 980 + Pertuzumab (*n* = 18) *n* (%)	Trastuzumab RP + Pertuzumab (*n* = 18) *n* (%)
Baseline		
Normal result	18 (100)	15 (83.3)
Abnormal result	0	3 (16.7)
Clinically significant	0	0
Not clinically significant	0	3 (16.7)
Day 1 at 4 h		
Normal result	17 (94.4)	14 (77.8)
Abnormal result	1 (5.6)	3 (16.7)
Clinically significant	0	0
Not clinically significant	1 (5.6)	3 (16.7)
Day 92/EOS		
Normal result	16 (88.9)	14 (77.8)
Abnormal result	2 (11.1)	4 (22.2)
Clinically significant	0	0
Not clinically significant	2 (11.1)	4 (22.2)

*ECG* electrocardiogram, *EOS* end-of-study

### Anti-drug antibodies

Immunogenicity was assessed at baseline prior to dosing and at day 92 (EOS). All subjects had an evaluable ADA result at baseline prior to dosing and at day 92 (EOS). The mean (± SD) incidence of pre-existing anti-ABP 980 or anti-trastuzumab RP binding ADAs was 0 (0.00). The incidence of neutralizing antibodies for both ABP 980 plus pertuzumab or trastuzumab RP plus pertuzumab were 0 (0.00) at baseline and EOS. Anti-pertuzumab antibodies were not assessed in the study.

### Pharmacokinetics

**Fig. 2:**
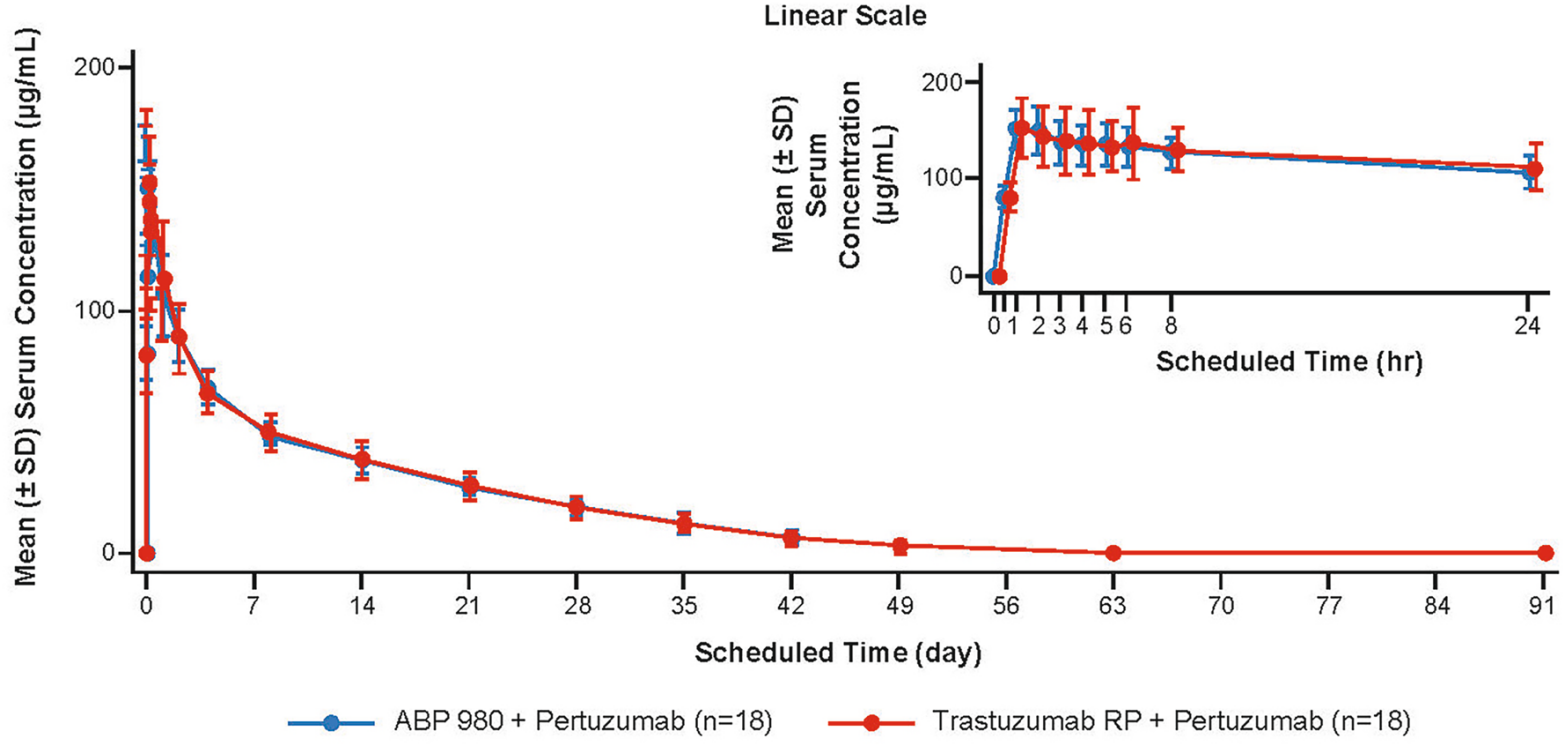
Serum concentrations (µg/mL; mean ± SD) of ABP 980 and trastuzumab RP over time

Mean (± SD) ABP 980 and trastuzumab RP serum concentration–time profiles through day 92 (EOS) are presented by treatment group for the PK Concentration Analysis Set in Fig. 2. Pertuzumab serum concentrations at each time point are summarized by treatment group for the PK Concentration Analysis Set in Table 4. Peak concentrations of ABP 980 and trastuzumab RP were observed approximately 1–2 h after the start of the infusion, with similar C_max_ and t_max_ in both treatment groups. The t_½_ of ABP 980 and trastuzumab RP was estimated to be between 5 and 7 days. Results show a high degree of similarity of overall PK profiles over the course of the study for the 2 treatment groups in terms of serum concentration–time profiles and all summary PK parameters.

**Table 4:** Summary of pharmacokinetic parameters

	ABP 980 + Pertuzumab (*n* = 18) Mean (± SD)^a^	Trastuzumab RP + Pertuzumab (*n* = 18) Mean (± SD)^a^
C_max_ (µg/mL)	160.1 (25.6)	158.6 (33.3)
C_last_ (µg/mL)	0.216 (0.098)	0.356 (0.257)
T_max_ (h)^﻿a^	1.03, 1.14, 6.00	1.03, 1.11, 6.00
AUC_inf_ (h*µg/mL)	36,147.8 (4666.8)	36,260.7 (6400.3)
AUC_last_ (h*µg/mL)	36,099.7 (4659.8)	36,198.1 (6406.0)
CL (L/h)	0.0124 (0.0016)	0.0128 (0.0018)
T_1/2_ (h)	165.59 (38.55)	119.94 (24.93)
λ_z_ (h)	0.0044 (0.0010)	0.0060 (0.0013)

^a^Data are presented as mean (± SD) except for T_max_ which is presented as minimum, median, maximum values

## Discussion

This study demonstrated the safety and tolerability of ABP 980 (6 mg/kg) and pertuzumab (420 mg) administered as an admixture in a single infusion bag given over 60 min. The frequency, type, and severity of AEs were similar between treatment groups with no clinically meaningful differences. Following the precedent of Wynne et al. based on the recommendation of pregnant women to avoid taking trastuzumab and the unknown effect of trastuzumab on reproductive capacity, this study only enrolled male subjects [13]. No new safety signals were identified, and the safety profiles were consistent with what is known for trastuzumab RP and pertuzumab in males. There were no meaningful differences observed in serum concentration–time profiles or PK parameters. No subject tested positive for binding or neutralizing ADAs to ABP 980 or trastuzumab RP during the study. These results demonstrate safety and tolerability of ABP 980 when co-infused with pertuzumab.

At the start of enrollment in the clinical study, it was observed during IV infusion that air bubbles formed in the IV lines causing the infusion pump to stop, which interrupted treatments to subjects. This issue was experienced by 5 of the first 6 subjects enrolled in the study and was not unique to either treatment. Enrollment was temporarily halted for an investigation to determine the cause of the air bubble formation. The results of the laboratory investigation demonstrated that air bubble formation in the IV lines was related to the temperature of the IV solution; the lower the temperature of the IV solution, the more likely that air bubbles formed in the IV-line drip chamber and tubing during infusion. On the basis of this observation, it was recommended to allow refrigerated IV solutions to warm up for approximately 3 h to room temperature prior to IV infusion to minimize the likelihood of air bubble formation in IV-line tubing. The Pharmacy Manual for the clinical study was revised to incorporate this recommendation and the initial 6 subjects were replaced and not included in any of the main analyses. The AE profile for these 6 subjects was consistent with that reported in the main analyses with no events greater than grade 2 in severity, no SAEs, and no events resulting in discontinuation from the study or a dose delay. The safety data from the 6 subjects did not change the overall safety conclusions of the study.

The safety of trastuzumab and pertuzumab administered as separate IV infusions has been well demonstrated. In a study published in 2010, patients with advanced HER2-positive breast cancer received trastuzumab weekly (4 mg/kg loading dose, then 2 mg/kg every week) or every 3 weeks (8 mg/kg loading dose, then 6 mg/kg every 3 weeks) and pertuzumab every 3 weeks (840 mg loading dose, then 420 mg every 3 weeks) [14]. In this study, the combination of pertuzumab and trastuzumab was effective and well tolerated in patients with metastatic HER2-positive breast cancer who had experienced progression during prior trastuzumab therapy. In a multicenter, open-label, phase 2 study published in 2012, women with HER2-positive breast cancer received trastuzumab (8 mg/kg loading dose, followed by 6 mg/kg every 3 weeks) plus docetaxel (75 mg/m^2^, escalating if tolerated, to 100 mg/m^2^ every 3 weeks; group A) or pertuzumab (loading dose 840 mg, followed by 420 mg every 3 weeks) and trastuzumab plus docetaxel (group B) or pertuzumab and trastuzumab (group C) or pertuzumab plus docetaxel (group D) [15]. Patients given pertuzumab and trastuzumab plus docetaxel (group B) had a significantly improved pathological complete response rate compared with those given trastuzumab plus docetaxel, without substantial differences in tolerability [15]. The safety results of this study are consistent with the results of the CLEOPATRA trial which demonstrated superior PFS and OS when pertuzumab was added to trastuzumab RP and docetaxel [16]. There were no unexpected toxicities in that study [17]. Also, in another phase 2 study, trastuzumab and pertuzumab administered in combination with paclitaxel was found to be well tolerated [16]. In the VELVET clinical trial of vinorelbine in combination with trastuzumab and pertuzumab for the first-line treatment of HER2-positive breast cancer, treatments were dosed sequentially (Cohort 1) and found to be reasonably well tolerated [18]; similar results were obtained when trastuzumab and pertuzumab were co-infused, followed by vinorelbine (Cohort 2) [19]. Lack of meaningful differences in outcome between the two cohorts supports administration of the combination of trastuzumab and pertuzumab in a single-bag infusion.

The mechanism by which combination dosing of trastuzumab RP and pertuzumab results in increased efficacy with no demonstrated impact on safety is through binding to distinct epitopes on the HER2 extracellular domain which results in a synergistic effect on the inhibition and survival of breast cancer cells [7, 8, 20, 21]. As trastuzumab RP binds to domain IV and pertuzumab binds to domain II of HER2, both therapeutics inhibit ligand-dependent HER2-HER3 heterodimerization and downstream signaling through a complementary mechanism. While the main mechanism of action of trastuzumab RP is thought to be through inhibition of signaling transduction, pertuzumab uniquely inhibits the potent HER2-HER3 dimerization [22, 23]. Xenograft tumor models have been used to further demonstrate enhanced antitumor activity with combination treatment resulting in tumor regression and inhibition of metastatic tumor spread in host animals, and with confirmation of a loss of cells expressing HER2 as opposed to downregulation of HER2 expression [8].

In conclusion, the results of the LAVENDER study presented here demonstrate that administration of the biosimilar ABP 980 plus pertuzumab in a single 60-min IV infusion is safe and feasible. The resulting PK of ABP 980 and trastuzumab RP were not affected by the co-infusion.

## Data Availability

There is a plan to share data. This may include de-identified individual patient data for variables necessary to address the specific research question in an approved data-sharing request; also related data dictionaries, study protocol, statistical analysis plan, informed consent form, and/or clinical study report. Data sharing requests relating to data in this manuscript will be considered after the publication date and 1) this product and indication (or other new use) have been granted marketing authorization in both the US and Europe, or 2) clinical development discontinues and the data will not be submitted to regulatory authorities. There is no end date for eligibility to submit a data sharing request for these data. Qualified researchers may submit a request containing the research objectives, the Amgen product(s) and Amgen study/studies in scope, endpoints/outcomes of interest, statistical analysis plan, data requirements, publication plan, and qualifications of the researcher(s). In general, Amgen does not grant external requests for individual patient data for the purpose of re-evaluating safety and efficacy issues already addressed in the product labeling. A committee of internal advisors reviews requests. If not approved, a Data Sharing Independent Review Panel may arbitrate and make the final decision. Requests that pose a potential conflict of interest or an actual or potential competitive risk may be declined at Amgen’s sole discretion and without further arbitration. Upon approval, information necessary to address the research question will be provided under the terms of a data sharing agreement. This may include anonymized individual patient data and/or available supporting documents, containing fragments of analysis code where provided in analysis specifications. Further details are available at the following: http://www.amgen.com/datasharing
